# Brain-inspired bodily self-perception model for robot rubber hand illusion

**DOI:** 10.1016/j.patter.2023.100888

**Published:** 2023-11-28

**Authors:** Yuxuan Zhao, Enmeng Lu, Yi Zeng

**Affiliations:** 1 Brain-inspired Cognitive Intelligence Lab, Institute of Automation, Chinese Academy of Sciences, Beijing 100190, China; 2 Center for Excellence in Brain Science and Intelligence Technology, Chinese Academy of Sciences, Shanghai 200031, China; 3 School of Future Technology, University of Chinese Academy of Sciences, Beijing 100049, China; 4 School of Artificial Intelligence, University of Chinese Academy of Sciences, Beijing 100049, China; 5 Center for Long-term Artificial Intelligence, Beijing, China

**Keywords:** bodily self-consciousness, rubber hand illusion, spatial statistics, brain-inspired model, multisensory integration, biological plausibility, computational mechanism, biological mechanism

## Abstract

The core of bodily self-consciousness involves perceiving ownership of one’s body. A central question is how body illusions like the rubber hand illusion (RHI) occur. Existing theoretical models still lack satisfying computational explanations from connectionist perspectives, especially for how the brain encodes body perception and generates illusions from neuronal interactions. Moreover, the integration of disability experiments is also neglected. Here, we integrate biological findings of bodily self-consciousness to propose a brain-inspired bodily self-perception model by which perceptions of bodily self are autonomously constructed without any supervision signals. We successfully validated the model with six RHI experiments and a disability experiment on an iCub humanoid robot and simulated environments. The results show that our model can not only well-replicate the behavioral and neural data of monkeys in biological experiments but also reasonably explain the causes and results of RHI at the neuronal level, thus contributing to the revelation of mechanisms underlying RHI.

## Highlights

• We propose a brain-inspired bodily self-perception model based on biological findings
• The model has more biological plausibility and interpretability compared with others
• The model can reproduce six RHI experiments and a disability experiment
• The model helps to reveal the computational and biological mechanisms behind the RHI

## The bigger picture

The rubber hand illusion (RHI) occurs when a participant’s hand and a model rubber hand are exposed to synchronized tactile and visual stimuli to the point where the participant will experience the illusion that the rubber hand becomes part of their own body. Questions remain about how these body illusions occur; particularly how different signals connect to encode the perception of one’s own body in the brain. Studying these kinds of perceptional illusions in robotic systems and with computational models can help us test our understanding of the underlying mechanisms. Our research takes inspiration from the existing biological findings, proposes a brain-inspired model of bodily self-perception, and validates it with multiple tasks in robots and simulated environments. This model helps to reveal the computational and biological mechanisms behind the RHI, ultimately establishing a solid foundation for further exploration into the machine implementation of bodily self-consciousness.

## Graphical abstract

**Figure:**
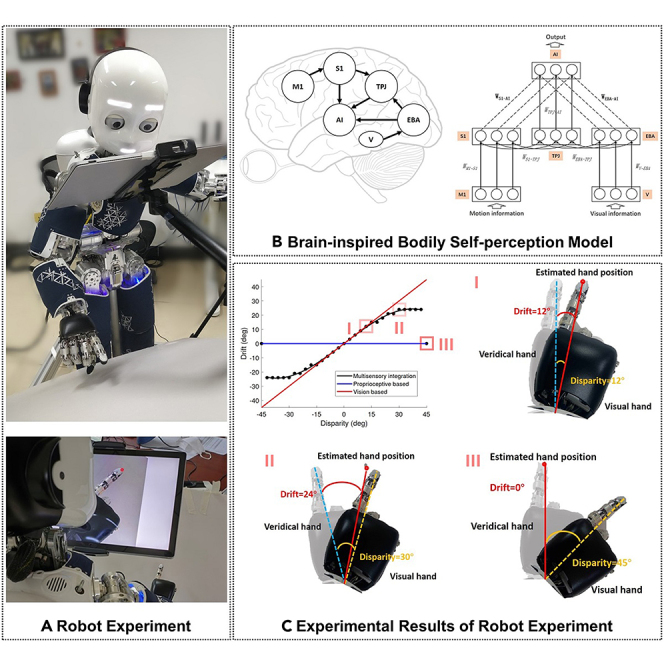

## Teaser

A central question in the study of bodily self-consciousness is how the rubber hand illusion (RHI) occurs. Here, the authors propose a brain-inspired bodily self-perception model for the RHI and validate it with various tasks in robotic and simulated environments. The model successfully replicates monkey behavioral and neural data from biological experiments, providing a reasonable explanation for the causes and results of the RHI at the neuronal level, contributing to our understanding of the underlying computational and biological mechanisms.

## Introduction

At the core of bodily self-consciousness is the perception of the ownership of one’s own body.^1^^,^^2^ In recent years, in order to understand more deeply how the brain encodes body ownership, researchers have attempted to establish a unified theoretical framework to explain the behavioral and neurophysiological phenomena in the coding of bodily self-consciousness from the perspectives of predictive coding and Bayesian causal inference. For these theoretical models, a core problem that needs to be explained is how body illusions such as the rubber hand illusion (RHI) occur. The RHI refers to the illusion that occurs when the participant’s rubber hand and the invisible real hand are presented with synchronized tactile and visual stimuli, in which the participant will have the illusion that the rubber hand seems to become his or her own hand.^3^

The prediction coding method constructs a model of the body by minimizing the errors between body perception and prediction.^4^ Hinz et al.^5^ regarded the RHI as a body estimation problem. They took humans and a multisensory robot that could perceive the proprioception, vision, and tactile information on its arm as participants and verified their model through a traditional rubber hand illusion experiment. The experiment showed that the proprioception drift was caused by prediction error fusion instead of hypothesis selection.

The active inference models can be regarded as an extension of predictive coding approaches. Rood et al.^6^ proposed a deep active inference model for studying the RHI. They modeled the rubber hand illusion experiment in a simulated environment. The results showed that their model produced perceptual and active patterns similar to those of humans during the experiment. Maselli et al.^7^ proposed an active inference model for arm perception and control, which integrates the intentional and conflict resolution imperatives. The imperative of intentional is used to control the achievement of external goals, and the imperative of conflict resolution is to avoid multisensory inconsistencies. The results revealed that intentional and conflict resolution imperatives were driven by different prediction errors.

The Bayesian causal inference model is extensively used in the theoretical modeling of multimodal integration and has been repeatedly verified at the behavioral and neuronal levels.^8^^,^^9^^,^^10^ The Bayesian causal inference model can well reproduce and explain a variety of RHI experiments. Samad et al.^11^ adopted the Bayesian causal inference model of multisensory perception and tested it through the rubber hand illusion on humans. The results showed that their model could reproduce the rubber hand illusion and suggested that the Bayesian causal inference dominates the perception of body ownership. Fang et al.^12^ presented a nonhuman primate version experiment of the rubber hand illusion. They conducted the experiments on human and monkey participants. They adopted the Bayesian causal inference (BCI) model to establish an objective and quantitative model for the body ownership of macaques. With the BCI model, they investigated the computational and biological mechanisms of body ownership in the macaque brain from monkey behavioral and neural data, respectively. The results show that the BCI model can fit the monkey behavioral and neuronal data well, helping to reveal a cortical representation of body ownership.

While existing theoretical models have provided explanations for the computational mechanisms underlying the RHI, primarily from the perspectives of predictive coding and BCI, a satisfactory explanation from a connectionist viewpoint remains elusive. Particularly, these models have not yet fully elucidated how the brain encodes the sense of bodily self-consciousness and generates the subjective experience of the RHI through the neuronal interactions within neural networks. Due to methodological constraints, many of these existing theoretical models have a restricted capacity to replicate a wide range of RHI experiments. Furthermore, these models rarely incorporate an assessment of how impairments in neural connectivity might affect their performance. Regarding this, we contend that evaluating the performance of computational models through disability experiments holds significant promise. Such experiments could shed light on the specific contributions of different modules within the models to uncover the computational and neural mechanisms underpinning the RHI. Just as similar brain-inspired computational models^13^ can inspire psychologists to design novel experimental paradigms to explore the biological mechanisms of the theory of mind,^14^ our results of disability experiments also have the potential to assist researchers in the cognitive field to further explore the biological mechanisms of the rubber hand illusion.

In this study, we accomplished the following. (1) We integrated the biological findings of bodily self-consciousness and constructed a brain-inspired bodily self-perception model. This model achieved association learning of proprioceptive and visual information, autonomously constructing the bodily self-perception model without any supervision signals. (2) We validated the model through six RHI experiments. The results indicated that the model reproduced these experiments simultaneously at both behavioral and neuronal scales. Moreover, the experimental results fitted well with the behavioral and neural data obtained from monkeys in biological experiments. (3) Additionally, we challenged the model using a disability experiment. The results suggested that the generation of the RHI resulted from the joint action of both the primary multisensory integration area, such as the temporo-parietal junction (TPJ), and the high-level multisensory integration area, such as the anterior insula (AI), with neither area being indispensable.

Our contributions are as follows. (1) We constructed a brain-inspired bodily self-perception model from the perspective of connectionist modeling, thereby enriching the spectrum of the theoretical models of bodily self-consciousness. (2) Compared with other computational models, our model exhibits greater biological plausibility and interpretability and replicates more experiments. It offers a reasonable explanation for the causes and results of the RHI at the neuronal level, thus contributing to revealing the computational and biological mechanisms underlying the occurrence of the RHI. (3) The results of our disability experiment also have the potential to assist researchers in the cognitive field to further explore the biological mechanisms of the RHI.

## Results

In this section, we introduce the brain-inspired bodily self-perception model, the design of the experiment, and the proprioceptive drift experiment results on the iCub humanoid robot and the results of proprioceptive drift, proprioceptive precision, appearance replacement, asynchronous, proprioception-only, vision-only, and disability experiments in the simulated environment.

### Brain-inspired bodily self-perception model

**Figure 1:**
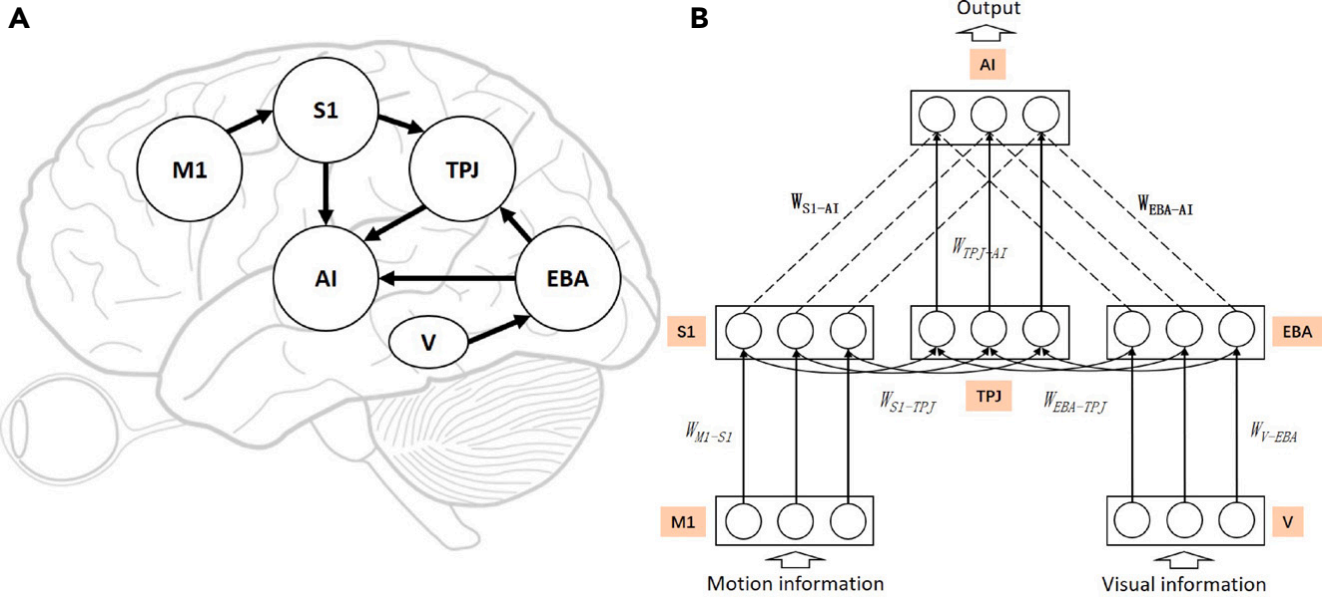
The architecture of the brain-inspired bodily self-perception model and its neural network architecture (A) Brain-inspired bodily self-perception model (including major functional brain areas, pathways, and their interactions); the arrow indicates the direction of information transmission. M1, primary motor cortex; S1, primary somatosensory cortex; TPJ, temporo-parietal junction; V, vision; EBA, extrastriate body area; AI, anterior insula. (B) The neural network architecture of the model. Brain areas are shown in orange, neurons in brain areas are shown as circles, and the connections between different brain areas are shown as black lines. The dashed lines denote excitatory or inhibitory synapses that are dependent on the results of synaptic plasticity (such as $W_{S1-AI}\;and\;W_{EBA-AI}$, in bold), and the solid lines indicate fixed synaptic weights (such as $W_{M1-S1},W_{S1-TPJ},W_{TPJ-AI},W_{V-EBA},W_{EBA-TPJ}$, in italics).

We integrated the biological findings of bodily self-perception into a model to construct a brain-inspired model for robot bodily self-perception; the architecture of the model is shown in Figure 1A.

We derived and designed our computational model to include multiple functional brain areas based on the current biological understanding of the functions of various brain areas involved in bodily self-perception. Specifically, the primary motor cortex (M1) is considered to encode the strength and orientation of motion and control motion execution.^15^^,^^16^ In our computational model, the M1 is used to encode the orientation of motion. The primary somatosensory cortex (S1) is considered for proprioception to perceive the limb position.^17^^,^^18^ In our computational model, the S1 receives stimuli from the M1 to perceive arm movement orientation. The extrastriate body area (EBA) is involved in the visual perception of the human body and body parts,^19^ and in the RHI experiment, it is proved to be directly involved in the processing of limb ownership.^20^ In our computational model, the EBA receives information from vision (V) and obtains finger orientation information. The TPJ integrates information from visual, auditory, and somatosensory systems, plays an important role in multisensory bodily processing,^21^^,^^22^ and has been reported to be activated in the RHI experiment.^23^^,^^24^ In our computational model, the TPJ receives information from the S1 and EBA and performs primary multisensory information integration. The AI is considered to be a primary region for processing interoceptive signals^1^ and multimodal sensory processing,^25^^,^^26^^,^^27^ and it has also been reported to be activated in the RHI experiment.^23^^,^^28^ In our computational model, the AI receives information from the S1, TPJ, and EBA; performs high-level multisensory information integration; and outputs the result.

Figure 1B shows the neural network architecture of the proposed model. This was a three-layer spiking neural network model with cross-layer connections. The M1 area generates information regarding the robot’s direction of motion, and the V area receives visual information regarding the robot. The AI area outputs the behavioral decision information of the robot based on the highest neuron firing rate. Unsupervised learning of the bodily self-perception model was realized through motor and visual information and tested using the RHI adapted from the macaque behavior experiment.^12^ More details can be found under experimental procedures.

### Experiments on the iCub robot

We chose the experimental paradigm of Fang et al.^12^ for robots. The classic RHI experiment is induced by touch and vision, whereas the RHI experiment conducted by Fang et al.^12^ on macaques and humans is induced by proprioception and vision, which requires lower hardware requirements for robots (without the need for tactile sensors). Most importantly, they provide objective and quantifiable behavioral experimental results, as well as neuronal scale experimental results, which can validate the effectiveness of our model from both behavioral and neuronal scales. The RHI experiment on the iCub robot can be seen in Video S1).

Video S1. Rubber hand illusion experiment on the iCub robot *Part I*: Experiment on the iCub robot (Multisensory integration - Visual dominance): When the hand rotation angle is small (small disparity angle), the proprioceptive drift is small. The robot mainly relies on visual information for decision-making.
*Part II*: Experiment on the iCub robot (Multisensory integration - Proprioception dominance): When the hand rotation angle is medium (medium disparity angle), the proprioceptive drift is medium. The robot mainly relies on proprioceptive information for decision-making.
*Part III*: Experiment on the iCub robot (Multisensory integration - Proprioception based): When the hand rotation angle is large (large disparity angle), the proprioceptive drift is zero. The robot completely relies on the proprioceptive information for decision-making.

#### Experimental design

**Figure 2:**
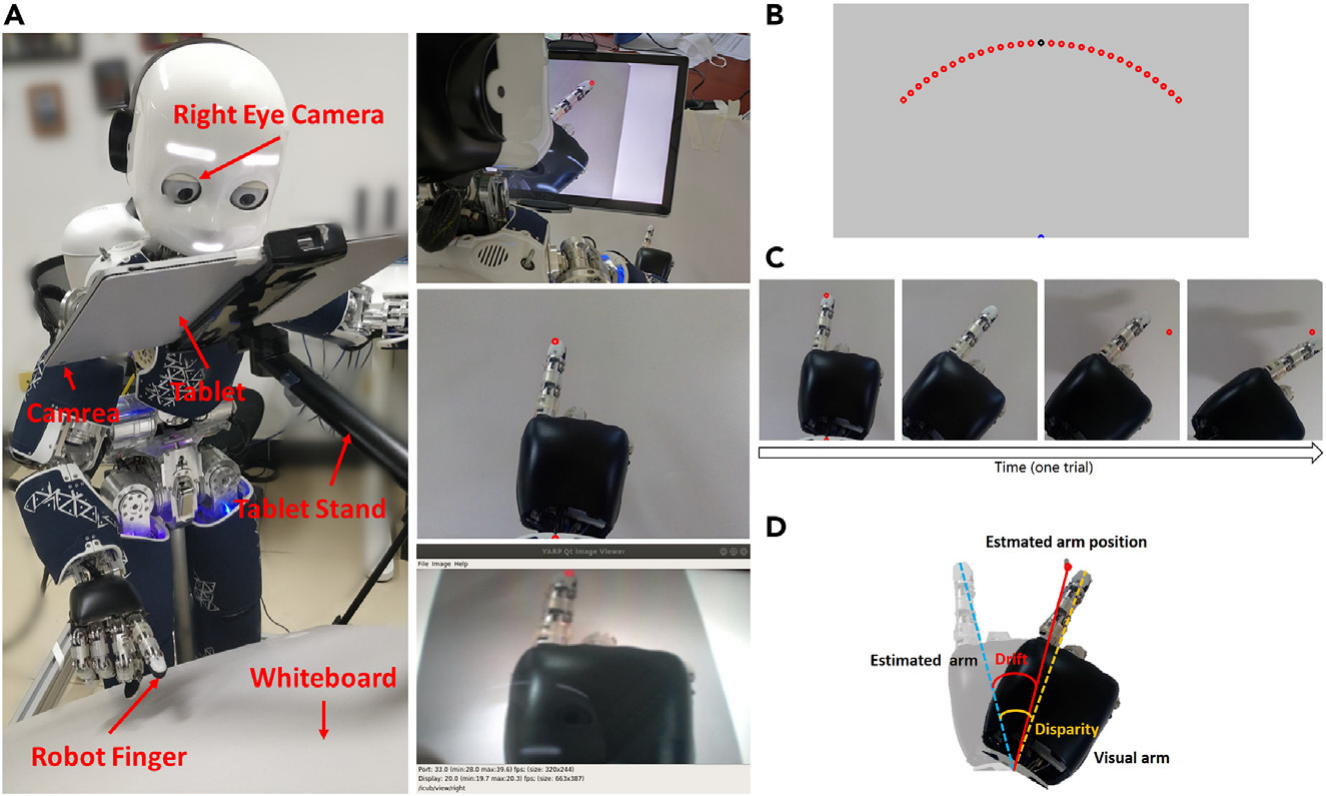
Overview of the robot experiment (A) Robot experiment settings. (B) Starting position and target. (C) Overview of one trial in the task. (D) Proprioceptive drift.

Figure 2A shows the experimental settings. Shown on the left is the equipment used in the robot experiment, including an iCub humanoid robot, a Surface Book 2 tablet with a camera on its back, a tablet stand, and white cardboard. The robot’s hand was placed between the tablet and white cardboard. The tablet served two functions: blocking the robot from seeing its own hand directly and capturing images of the robot’s hand through the back camera, rotating the image, and displaying the processed image in front of the robot’s own eye camera (right eye camera). The tablet stand was used to support and hold the tablet in a desired position. The white cardboard blocks sundries to make the background as clean as possible, thereby reducing the difficulty of image recognition and the possibility of incorporating other intrusive visual cues. The equipment settings ensured that the robot could only perceive its visual hand through the tablet display using its own eye camera. Shown on the right, from top to bottom, are a scene from the robot experiment in the right rear view, an image displayed by the tablet, and an image perceived by the robot using its right eye camera.

Figure 2B shows the starting position and the target during the robot experiment. All points in the experiment are colored red, and we have marked the initial positions as blue and black points for ease of illustration. The robot first placed its wrist on the blue dot below the image and its finger on the black dot. The red dots represent the targets to which the robot needed to point during the experiment. These targets were distributed on a circular arc, with the blue dot as the circular dot (the position of the robot wrist) and the distance between the blue and black dots as the radius (the distance between the robot’s wrist and its fingertip). The point directly in front of the circular dot is $0^{\circ }$, ranging from $-45^{\circ }$ to $45^{\circ }$, and the interval is $15^{\circ }$. There are nine target points in total.

Figure 2C shows an overview of one-task trial. (1) The robot places its hand at the starting position. (2) The point at the starting position disappears, and the visual hand rotates at a random angle. Considering the motion range and precision of the robot, the rotation angle ranges from $-36^{\circ }$ to $36^{\circ }$, and the interval is $6^{\circ }$, including two angles $-45^{\circ }$ and $45^{\circ }$. There are 15 rotation angles in total. (3) The target is displayed. (4) Based on multisensory integration by vision and proprioception, the robot makes behavioral decisions and points to the target.

Figure 2D shows the measurement method of proprioceptive drift. The dark hand is the visual hand, and the light hand is the veridical hand. The red target point represents the position of the hand as estimated by the robot, while the veridical hand represents the position of the proprioceptive hand. Therefore, the angle between the robot’s veridical hand and the target point is the proprioceptive drift in the RHI; that is, the angle between the blue dotted line and the red solid line. The angle between the blue and orange dotted lines represents the disparity between the veridical and visual hand.

#### Experimental results

**Figure 3:**
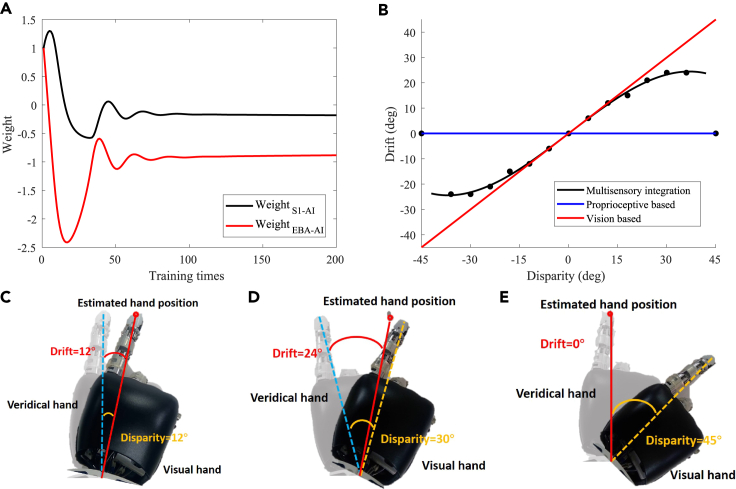
Experimental results of robot experiment (A) Weight changing. (B) Behavioral result. The black dots represent the results of behavioral experiments, and the black line represents the curve-fitting results of the behavioral data (multisensory integration). The red line represents the proprioceptive drift that completely depends on the visual information (vision based). The blue line represents the proprioceptive drift that completely depends on the proprioceptive information (proprioceptive based). The horizontal axis is the disparity degree between the visual hand and the veridical hand, and the vertical axis is the degree of proprioceptive drift. (C) Small disparity angle. (D) Medium disparity angle. (E) Large disparity angle.

Before the RHI experiment, the robot was required to construct a bodily self-perception model through training. During the training process, the robot directly observed its veridical hand and trained it through random movements. The training process did not require any supervised signals. Figure 3A shows the synaptic weights between the S1 and AI as well as the EBA and AI after training, both of which changed from excitatory to inhibitory connections. It also shows that the intensity of inhibitory connection from vision (EBA-AI) was higher than that from proprioception (S1-AI).

Figure 3B shows the behavioral results of the robot experiment. The red diagonal line represents the proprioceptive drift that completely depends on visual information (vision based); that is, when there is only visual information input, the robot completely relies on visual information for decision-making, so the proprioceptive drift increases with the increase of visual disparity. The blue horizontal line represents the proprioceptive drift that completely depends on the proprioceptive information (proprioceptive based); that is, when there is only proprioceptive information input, the robot completely relies on proprioception information for decision-making, so the proprioceptive drift is consistently zero. The black dots represent the results of behavioral experiments, and the black line represents the curve-fitting results of the behavioral data (multisensory integration). Thus, when the black line is closer to the red diagonal line, it indicates that the robot relies more on vision for decision-making. When the black line is closer to the blue horizontal line, it indicates that the robot relies more on proprioception for decision-making. (1) When the hand rotation angle was small (small disparity angle), the proprioceptive drift was small. With the increase in the disparity angle, the proprioceptive drift increased, and the black dots were closer to the red diagonal line, indicating that the robot relied mainly on visual information for decision-making. (2) When the hand rotation angle was medium (medium disparity angle), the proprioceptive drift was medium. With the increase of the disparity angle, the proprioceptive drift increased slowly or remained unchanged, indicating that the role of visual information in decision-making was weakened, while the role of proprioceptive information in decision-making was enhanced. The robot mainly relies on proprioceptive information for decision-making. (3) When the hand rotation angle was large (large disparity angle), the proprioceptive drift was zero. With the increase in the disparity angle, the proprioception deviation did not change, and the black dots were closer to the blue horizontal line, indicating that the robot completely relied on the proprioceptive information for decision-making.

Figure 3C shows the results of the behavior at a small disparity angle. The disparity angle was $12^{\circ }$, and the proprioceptive drift was $12^{\circ }$. Figure 3D shows the results of the behavior at a medium disparity angle. The disparity angle was $30^{\circ }$, and the proprioceptive drift was $24^{\circ }$. Figure 3E shows the results of the behavior at a large disparity angle. The disparity angle was $45^{\circ }$, and the proprioceptive drift was $0^{\circ }$.

### Experiments in the simulated environment

#### Proprioceptive drift and proprioceptive precision experiments

**Figure 4:**
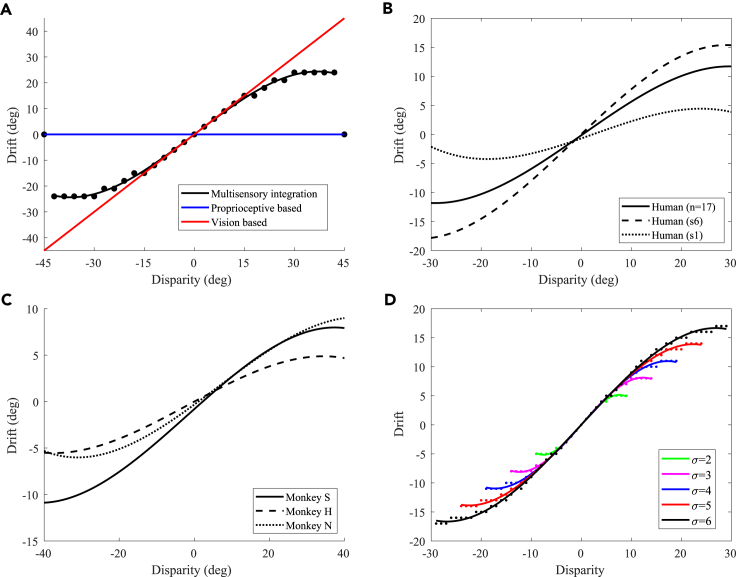
Experimental results of the proprioceptive drift experiment and proprioceptive precision experiment (A) Proprioceptive drift experiment result. (B) Proprioceptive drifts of human participants.^12^ We reference the experimental results of Fang et al.^12^ and plot the fitting curve of proprioceptive drift results in human participant experiments. The solid line represents the fitted curve of the average proprioceptive drift values from 17 human participants in their experiment, the dashed line represents the fitted curve of proprioceptive drift for the sixth subject (S6) in the experiment, and the dotted line represents the fitted curve of proprioceptive drift for the first subject (S1) in the experiment. (C) Proprioceptive drifts of macaque monkeys.^12^ We reference the experimental results of Fang et al.^12^ and plot the fitting curve of proprioceptive drift results in macaque monkey experiments. The solid line, dashed line, and dotted line represent the fitted curve of proprioceptive drift for monkey S, monkey H, and monkey N in the experiment. (D) Proprioceptive precision experiment result.

In the simulated environment, the rotation angle ranges from $-60^{\circ }$ to $60^{\circ }$, and the interval is $3^{\circ }$. The result of the proprioceptive drift experiment is shown in Figure 4A, which is similar to the results of the RHI behavior experiment in macaques and humans.^12^ Here we refer to the experimental results obtained by Fang et al.^12^ and plotted the fitting curve of the proprioceptive drift results for human participant and macaque monkey experiments, as shown in Figures 4B and 4C. The behavior experiment results in Fang et al.^12^ show that the proprioceptive drifts increased for small levels of disparity, while it plateaus or even decreases when the disparity exceeds $20^{\circ }$. In our experiment, the proprioceptive drift increases rapidly when the disparity was less than $21^{\circ }$ and became flatter when the disparity was greater than $21^{\circ }$. The results showed that, when the disparity between the visual hand and the robot’s veridical hand was small, the hand position perceived by the robot was closer to the position of the visual hand, whereas when the disparity was large, the robot’s perception of the hand position was more dependent on the hand position of proprioception.

A recent study has shown that proprioceptive precision may influence the proneness to the RHI, but there are few relevant studies.^29^(“Proprioceptive accuracy” is a specialized term used by psychologists, referencing the usage found in Horváth et al.^29^ Considering the variations across different disciplinary backgrounds and to enhance clarity and accuracy in the description, we consider “proprioceptive precision” to be more accurate from a computational perspective, aligning more closely with the customary articulation of “accuracy” and “precision” concepts in machine learning.) In the proposed model, the precision of the robot’s proprioceptive perception can be achieved by controlling the standard deviation of the receptive field of the neuron model. The larger the standard deviation, the wider the receptive field range and the lower the accuracy. In contrast, the smaller the standard deviation, the narrower the receptive field range and the higher the accuracy. Therefore, we tested the effect of proprioceptive precision on the RHI by controlling the standard deviation of the neuron receptive field; the result is shown in Figure 4D. This indicates that, when the proprioceptive precision was high (that is, the standard deviation of the receptive field was small), the robot could only produce illusions within a small disparity range, and when the proprioceptive precision was low (that is, the standard deviation of the receptive field was larger), the robot could produce illusions within a large disparity range. That is, the experimental results of the model prove that a lower proprioceptive precision is more likely to induce the RHI, while a higher proprioceptive precision is more difficult to induce. This conclusion is consistent with the findings of recent studies.^29^^,^^30^ Note that, to make the experimental results more intuitive, we unified the behavioral results of different proprioceptive accuracies into a scale range.

In the proprioceptive drift experiment, the robot’s behavior was influenced by the dynamic changes of the neurons’ firing rate in the model. In the proposed model, multisensory integration takes place in two areas: the TPJ and AI. The TPJ is a low-level area of multisensory integration that initially integrates proprioceptive and visual information. For robots, there is a sequential relationship as follows: motion commands are issued (stimulating neurons in the M1 to fire), the robot’s fingers perform the motion, and the camera detects the finger motion (stimulating neurons in V to fire). Multisensory integration in the TPJ occurs at the moment when neurons in both the S1 and EBA are firing. When the camera detects finger movement, it stimulates the neurons in V to fire and transmits it to the EBA. Neurons in the EBA are stimulated to fire, and the firing rate increases. At this time, the stimulation in the M1 disappears, and the firing rate of the neurons in the S1 decreases. That is, when the TPJ integrates proprioceptive information in the S1 and visual information in the EBA, the firing rate of neurons in the S1 decreases, while the firing rate of neurons in the EBA increases. Therefore, the TPJ is affected more by visual information during multisensory integration.

The AI is a high-level area of multisensory integration. It integrates the proprioceptive information from the S1, visual information from the EBA, and initial proprioceptive-visual integration information from the TPJ to achieve the final integration of multisensory information, and the integration result affects the behavior of the robot. After training, $W_{S1-AI}$ and $W_{EBA-AI}$ changed from the initial excitatory connections to inhibitory connections. The inhibitory strength of $W_{EBA-AI}$ was greater than that of $W_{S1-AI}$, indicating that the AI had a strong inhibitory effect on visual information during multisensory integration (Figure 3A).

**Figure 5:**
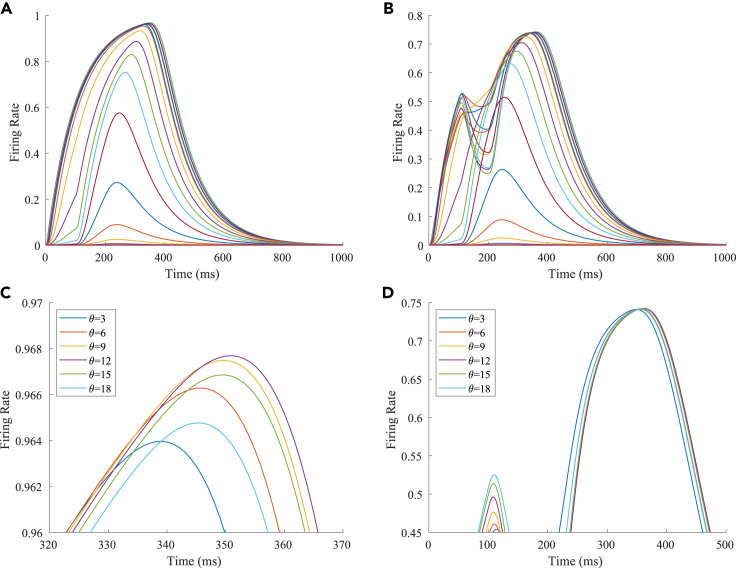
The firing rate of the neurons in the TPJ and AI in the proprioceptive drift experiment (multisensory integration-visual dominance) The proprioceptive perception is $0^{\circ }$, and the visual disparity is $12^{\circ }$. The proprioception drift is $12^{\circ }$ after multisensory integration. (A and B) The firing rate of the neurons in the TPJ and AI. (C and D) The results of local amplification near the maximum peak firing rates of the neurons in the TPJ and AI. Here, the six neurons with the highest firing rates are selected, and the dynamic changes of these neurons are plotted.

The firing rates of the TPJ and AI in the proprioceptive drift experiment (multisensory integration-visual dominance) are shown in Figure 5. When visual disparity is small, the receptive field overlap of proprioceptive and visual information is huge; therefore, the TPJ area presents a single-peak, visual-information-dominated multisensory integration result. The results of multisensory integration when the proprioceptive perception was $0^{\circ }$ and the visual disparity was $12^{\circ }$ are shown in Figures 5A and 5C. Figure 5C shows the dynamic changes of the six neurons with the highest firing rate in the TPJ. The firing rates from high to low are $12^{\circ },9^{\circ },15^{\circ },6^{\circ },18^{\circ },3^{\circ }$, and the initial integration result in the TPJ is $12^{\circ }$. The AI area presents multisensory integration results of the bimodal peaks (Figures 5B and 5D). The anterior peak is mainly inhibited by neurons near the proprioceptive perception $0^{\circ }$ and the visual perception $12^{\circ }$. The firing rates from high to low were $18^{\circ },15^{\circ },12^{\circ },9^{\circ },6^{\circ },3^{\circ }$, and the result of anterior peak integration was $18^{\circ }$. In the posterior peak, the inhibition intensity decreased with the disappearance of the proprioceptive and visual stimulation, and the firing rates were $12^{\circ },9^{\circ },15^{\circ },6^{\circ },18^{\circ },3^{\circ }$ from high to low. The firing rate of neurons shows the posterior peak is higher than that of the anterior peak, and the final integration result in the AI was $12^{\circ }$.

**Figure 6:**
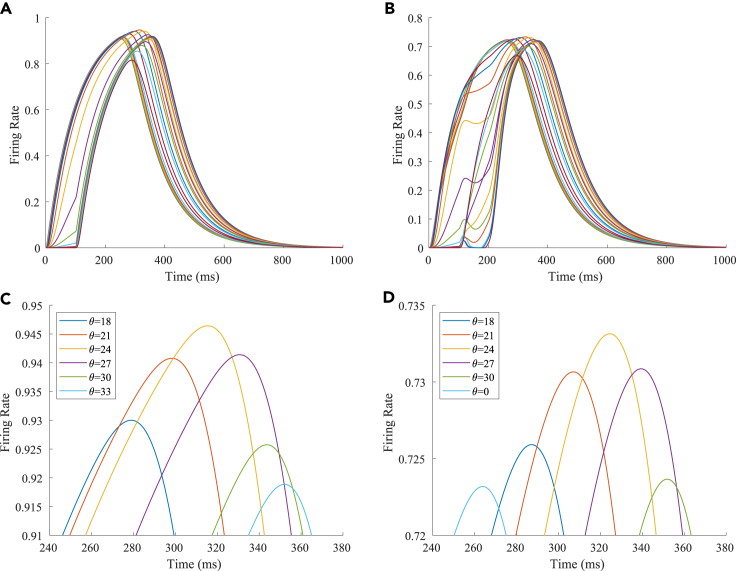
The firing rate of the neurons in the TPJ and AI in the proprioceptive drift experiment (multisensory integration-proprioception dominance) The proprioceptive perception is $0^{\circ }$, and the visual disparity is $39^{\circ }$. The proprioception drift is $24^{\circ }$ after multisensory integration. (A and B) The firing rate of the neurons in the TPJ and AI. (C and D) The results of local amplification near the maximum peak firing rates of the neurons in the TPJ and AI. Here, the six neurons with the highest firing rates are selected, and the dynamic changes of these neurons are plotted.

The firing rates of the TPJ and AI in the proprioceptive drift experiment (multisensory integration-proprioception dominance) are shown in Figure 6. When visual disparity is moderate, the information integration in the TPJ mainly occurs at the location where the proprioceptive information and visual information receptive fields overlap. The TPJ area presented a multipeak, proprioceptive-information-dominated multisensory integration result. The results of multisensory integration when the proprioceptive perception was $0^{\circ }$ and the visual disparity was $39^{\circ }$ are shown in Figures 6A and 6C. Figure 6C shows the dynamic changes of the six neurons with the highest firing rate in the TPJ. The firing rates from high to low were $24^{\circ },27^{\circ },21^{\circ },18^{\circ },30^{\circ },33^{\circ }$, and the initial integration result in the TPJ was $24^{\circ }$. The AI area presented multipeak, multisensory integration results (Figures 6B and 6D). Figure 6B shows the dynamic changes of all neurons. Figure 6D shows the dynamic changes of the six neurons with the highest firing rate in the AI. The firing rates from high to low were $24^{\circ },27^{\circ },21^{\circ },18^{\circ },30^{\circ },0^{\circ }$. The final integration result in the AI was $24^{\circ }$.

**Figure 7:**
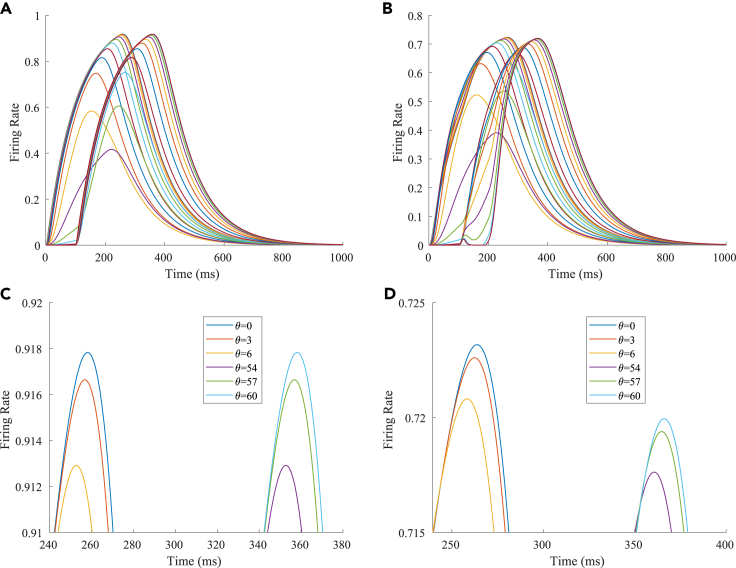
The firing rate of the TPJ and AI in the proprioceptive drift experiment (multisensory integration-proprioception based) The proprioceptive perception is $0^{\circ }$, and the visual disparity is $60^{\circ }$. The proprioception drift is $0^{\circ }$ after multisensory integration. (A and B) The firing rate of the neurons in the TPJ and AI. (C and D) The results of local amplification near the maximum peak firing rates of the neurons in the TPJ and AI. Here, the six neurons with the highest firing rates are selected, and the dynamic changes of these neurons are plotted.

The firing rates of the TPJ and AI in the proprioceptive drift experiment (multisensory integration-proprioception based) are shown in Figure 7. When the visual disparity is large, the overlapping area of proprioceptive neurons and visual neurons’ receptive fields is small, and the integration of the TPJ is weak. Therefore, the TPJ presented complete doublet peak results, with the anterior peak dominated by proprioceptive information and the posterior peak dominated by visual information. The results of multisensory integration when the proprioceptive perception was $0^{\circ }$ and the visual disparity was $60^{\circ }$ are shown in Figures 7A and 7C. Figure 7C shows the dynamic changes of the six neurons with the highest firing rate in the TPJ. It shows that the anterior peak presented the integration result dependent on proprioception information, and the firing rates from high to low were $0^{\circ },3^{\circ },6^{\circ }$. The posterior peak presented the integration result dependent on visual information, and the firing rates from high to low were $60^{\circ },57^{\circ },54^{\circ }$. The firing rates of the six neurons were $60^{\circ },0^{\circ },57^{\circ },3^{\circ },54^{\circ },6^{\circ }$ from high to low, and the initial integration result in TPJ was $60^{\circ }$. The AI area presents doublet-peak multisensory integration results (Figures 7B and 7D). Because the inhibition weight of visual connections ($W_{EBA-AI}$) in the AI area was greater than that of proprioception ($W_{S1-AI}$), the anterior peak that relies on proprioception is used as the final output. The firing rates from high to low were $0^{\circ },3^{\circ },6^{\circ },60^{\circ },57^{\circ },54^{\circ }$. The final integration result in the AI was $0^{\circ }$.

#### Appearance replacement and asynchronous experiments

**Figure 8:**
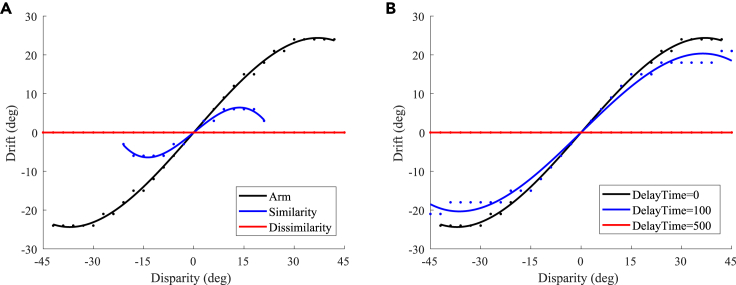
The results of the appearance replacement experiment and asynchronous experiment (A) Appearance replacement experiment. (B) Asynchronous experiment.

The result of the appearance replacement experiment is shown in Figure 8A, which is similar to the results of the prior knowledge of body representation behavior experiment (that is, replacing the visual hand in the RHI with a wooden block) in macaques and humans.^12^ The experimental result in Fang et al.^12^ shows that the proprioceptive drifts under the wood condition were significantly reduced compared with those under the visual hand condition. In our experiment, in the case of high similarity (that is, the hand in the robot’s field of vision was similar to its own hand), the proprioceptive drift of the robot was significantly smaller than that of the own visual hand. In addition, the range of visual disparity that can induce the robot’s RHI is narrower. When the disparity exceeded $21^{\circ }$, the robot’s proprioceptive drift was $0^{\circ }$, and the RHI disappeared. In the case of dissimilarity (that is, the hand in the robot’s field of vision is completely different from its own hand), the robot’s proprioceptive drift is $0^{\circ }$, which indicates that the robot does not consider the hand in the field of view as its own. At the neuronal scale, the response of neurons in the EBA area to a similar hand is lower than that of their own hand, which leads to the weak contribution of visual information for multisensory integration, with the TPJ and AI relying more on proprioceptive information for integration; the neurons in the EBA area respond much less to dissimilar hands than their own hand, further weakening the contribution of visual information for multisensory integration in the TPJ and AI areas.

Other studies^31^^,^^32^ have shown that the majority of participants would not experience the RHI if the asynchrony was greater than 500 or 300 ms. Here we tested the effect of synchrony on the RHI. The result of the asynchronous experiment is shown in Figure 8B. When the delay time was 100 ms, the proprioceptive drift of the robot was smaller than that of the own visual hand. When the delay time was 500 ms, the robot’s proprioceptive drift was $0^{\circ }$, indicating that the robot did not conclude that the hand in the field of view was its own. At the neuronal scale, the delayed presentation of visual information weakens the contribution of visual information to multisensory integration, and a large delay results in the inability to integrate proprioceptive and visual information within the effective firing time window of neurons.

#### Proprioception-only and vision-only experiments

When there is only proprioceptive information (that is, when the robot only moves its own hand, and no hand is observed in the field of vision), the AI neuron is fired, indicating that the robot concludes that the moving hand belongs to itself. When there is only visual information (that is, the robot’s own hand does not move, but the hand movement can be seen in the field of vision), the neuron in AI is not fired, indicating that the robot does not think that the moving hand belongs to itself. At the neuronal scale, the main reason for this result was that the inhibition intensity of visual information in the AI was stronger than that of proprioceptive information.

#### Disability experiment

We explored the effects of TPJ disability and AI disability on the RHI by setting synaptic weights between different brain areas. Consider the following proprioceptive precision experiment as an example.

**Figure 9:**
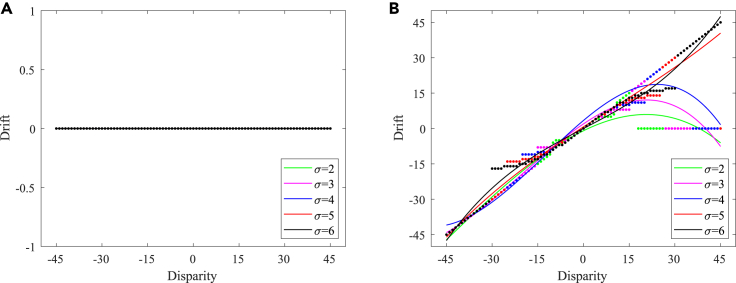
The results of the disability experiment (A) TPJ disability experiment. (B) AI disability experiment.

In the TPJ disability experiment, we set $W_{S1-TPJ}$ and $W_{EBA-TPJ}$ as 0. Multisensory information was integrated by the AI only, and the TPJ no longer integrated any information. The experimental result of TPJ disability with different proprioceptive precision after training and testing is shown in Figure 9A. This result indicates that, when the TPJ is completely disabled, multisensory information integration by the AI alone cannot induce the RHI. In addition, we simulated the impact of varying extents of TPJ disability on the RHI by setting different synaptic weights. The experimental results show that, when the extent of TPJ disability is low (that is, the values of $W_{S1-TPJ}$ and $W_{EBA-TPJ}$ are large), TPJ is still able to integrate multisensory information, and the robot can be induced to have the RHI. But the proprioceptive drift is smaller, and the range of visual disparity that can induce the robot’s RHI is narrower. As the extent of disability increases (that is, the values of $W_{S1-TPJ}$ and $W_{EBA-TPJ}$ decrease), the RHI becomes more difficult to induce. The experimental results are consistent with the behavioral experiment results when using transcranial magnetic stimulation over the TPJ to reduce the extent of the RHI.^33^

In the AI disability experiment, we set $W_{S1-AI}$, $W_{TPJ-AI}$, and $W_{EBA-AI}$ as 0. Multisensory information is integrated by the TPJ, and the AI no longer integrated any information. The experimental result of AI disability with different proprioceptive precision after training and testing is shown in Figure 9B. As seen in Figure 9B, although the robot is induced with the RHI at partial visual deflection angles, the AI disability robot mainly relies on vision for decision-making at most visual deflection angles. That is, the AI disability cannot effectively induce the RHI.

The results of the disability experiment indicate that the generation of the RHI is the result of the joint action of the primary multisensory integration area (e.g., the TPJ) and the high-level multisensory integration area (e.g., the AI), and neither is indispensable.

### Comparison with other models

Computational models of self-body representation, especially those that can reproduce and explain the RHI, have been less studied. Researchers have proposed theoretical models of body representation mainly from the perspectives of predictive coding and BCI.^34^ Table 1 lists a comparison of results with those of the other models.

**Table 1:** Comparison with other models

Experiment	(5)	(35)	(6)	(7)	(11)	(12)	Our model
Proprioceptive drift	O	O	O	O	O	O	O
Proprioceptive precision	–	–	–	–	–	–	O
Appearance replacement	–	–	–	–	–	O	O
Asynchronous	–	–	O	–	O	–	O
Proprioception only	–	–	–	–	O	–	O
Vision only	–	–	–	–	O	–	O
Disability	–	–	–	–	–	–	O
Model	PC	PC	deep AIF	AIF	BCI	BCI	brain-self
Participant	H, R	R	S	H	H	M, H	R, S

Model: PC, predictive coding; AIF, active inference model; BCI, Bayesian causal inference; brain-self, brain-inspired bodily self-perception; H, human; R, robot; S, simulated environment; M, monkey. O represents that the model could reproduce the correlation feature, and *–* represents that it is not mentioned.

The core idea of the predictive coding approaches^5^^,^^35^ is to refine the body estimation through the minimization of the errors between perception and prediction. The active inference models^6^^,^^7^ can be regarded as an extension of predictive coding approaches. These methods can well reproduce and explain the proprioceptive drift experiment and have been verified in humans, robots, and a simulated environment. However, these studies did not involve various experiments of the RHI, nor could they explain the specific computational mechanism of individual neurons and population neurons.

The BCI model is extensively used in the theoretical modeling of multimodal integration and has been repeatedly verified at the behavioral and neuronal levels. The BCI model can well reproduce and explain a variety of RHI experiments.^11^^,^^12^ However, in the RHI experiment, most of the BCI models have problems similar to the predictive coding approaches, such as the explanatory scale remaining at the behavioral scale, and this also does not explain how the RHI is generated from the neuron scale. In addition, there are also some studies on neural network modeling, although they mainly focus on multimodal integration and do not involve RHI experiments.^36^

Compared with these models, we built a brain-inspired bodily self-perception model from the perspective of brain-inspired computing. The proposed model can not only reproduce as many as six experiments of the RHI but also reasonably explain the RHI from the neuron scale at the same time, which helps reveal the computational and neural mechanisms of the RHI. The computational model of the disability experiment demonstrated that the RHI cannot be induced without the TPJ, which performs primary multisensory integration, and the AI, which performs high-level multisensory integration.

## Discussion

In this study, we integrated the biological findings of bodily self-consciousness and constructed a brain-inspired bodily self-perception model that can construct bodily self-perception autonomously without any supervision signals. It can reproduce six RHI experiments and reasonably explain the causes and results of the RHI at the neuron scale. Compared with other models, the proposed model explains the computational mechanism whereby the brain encodes bodily self-consciousness and how the body illusion we subjectively perceive is generated by neural networks. Especially, the experimental results of this model can well fit the behavioral and neural data from monkeys in biological experiments. This model is helpful to reveal the computational and neural mechanism of the RHI.

In the biological experiment of the rubber hand, Fang et al.^12^ recorded the firing rate of neurons in the premotor cortex of two monkeys in the behavioral task. They defined two types of neurons: integration neurons and segregation neurons. When a neuron’s firing rate under the visual-proprioceptive congruent task is greater than that under the proprioception-only task, it indicates that the neuron prefers to integrate visual and proprioception information, which they define as “integration neuron.” Conversely, when a neuron’s firing rate under the proprioception-only task is greater than that under the visual-proprioceptive congruent task, it means that the neuron prefers only proprioception information, which they define as “segregation neuron.” By analyzing the firing rates of neurons in different tasks, they found that the firing rates of integration neurons decreased with increasing visual disparity during the target-holding period and that the firing rates of segregation neurons increased with increasing visual disparity during the preparation period.

**Figure 10:**
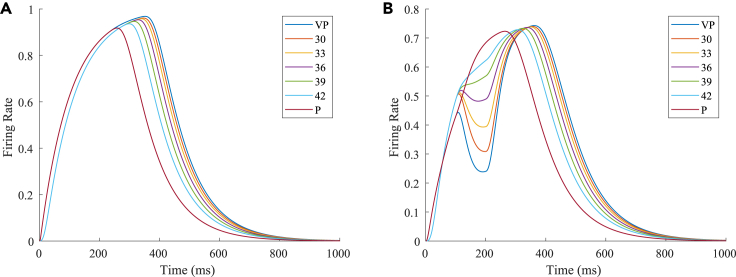
The dynamic changes of neuronal firing rates in the TPJ and AI areas in different tasks (A) TPJ area. (B) AI area.

In our computational model, neurons in the TPJ and AI areas exhibited similar properties. Figure 10 shows the dynamic changes of neuronal firing rates in the TPJ and AI areas in different tasks. To make the comparison of results more intuitive and significant, the results of the visual-proprioceptive (VP) congruent condition, proprioception-only (P) condition and part of VP conflict (VPC) conditions were selected for display. The VPC conditions include the results when proprioception was $0^{\circ }$ and visual deflections were $30^{\circ },33^{\circ },36^{\circ },39^{\circ },42^{\circ }$, respectively. Clearly, neurons in the TPJ area consistently exhibited integration properties. With the increase in visual disparity, the integration intensity of neurons in the TPJ becomes weaker, and the firing rate of neurons becomes lower. Neurons in the AI region exhibit the properties of separation and integration at different stages. In the early stage (approximately 200 ms), the neurons in the AI area exhibited separation properties, and the firing rate of neurons increased with the increase in visual disparity. In the later stage (approximately 400 ms), the neurons in the AI area exhibited integration properties, and the firing rate of neurons decreased with the increase in visual disparity.

The reason for the integration effect in the TPJ is that, when the visual disparity is small, the receptive field overlap of proprioceptive information and visual information is huge; therefore, the integration effect is strong, and the firing rate of neurons is high; when the visual disparity is huge, the receptive field overlap of proprioceptive information and visual information is small, so the integration effect is weak, and the firing rate of neurons is low.

The AI is a high-level area of multisensory integration that simultaneously receives an excitatory stimulus (proprioception-visual integration information) from the TPJ and an inhibitory stimulus from the S1 (proprioception information) and EBA (visual information). In the separation stage, the firing rate of neurons was highest in the P task and the lowest in the VP congruent task, and in the VPC task, the firing rates of the neurons increased with the increase in visual disparity. The reason is that, when the proprioceptive stimulation disappears, the firing rate of neurons in the S1 decreases; when the visual stimulation appears, the firing rate of neurons in the EBA increases, and the inhibitory effect of information from the EBA on information from the TPJ in the AI increases. In the P task, the firing rate of neurons in the AI was highest due to the absence of inhibition from the EBA. In the VP congruent task, the receptive fields of proprioceptive and visual information overlap completely; the inhibitory effect of the EBA on the TPJ was strongest in the AI, so the firing rate of neurons in the AI was the lowest. In the VPC task, the information integration in the TPJ mainly occurs at the location where the proprioceptive and visual information receptive fields overlap. With the increase in visual disparity, the inhibition of the EBA on the integration information in the TPJ decreases, leading to the increase in the firing rate of neurons in the AI. In the integration stage, the proprioceptive and visual stimulation disappears, and their firing rates decrease. Due to the cumulative effect, the firing rate of neurons in the TPJ continued to increase, and the inhibition of the S1 and EBA in the AI on the TPJ decreased, highlighting the integration effect of the TPJ.

## Experimental procedures

### Resource availability

#### Lead contact

Further information and requests for resources should be directed to and will be fulfilled by the lead contact, Dr. Yi Zeng (yi.zeng@ia.ac.cn).

#### Materials availability

This study did not generate new unique materials.

#### Data and code availability

All original code has been deposited at GitHub under https://github.com/Brain-Cog-Lab/RHI and at Zenodo under https://doi.org/10.5281/zenodo.8385850 and is publicly available as of the date of publication. The data used in this article are available from the code, and readers can also contact the lead contact for requests.

### Neuron model

The brain-inspired bodily self-perception model is shown in Figure 1.

**Figure 11:**
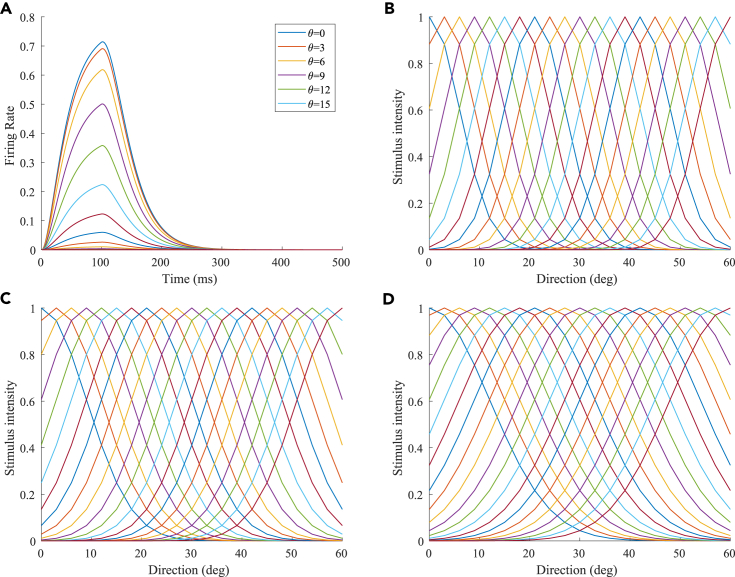
The characteristic behaviors of neurons (A) The firing rate of the neuron that represents $0^{\circ }$ when receiving $0^{\circ }$ stimulation; the stimulation is presented at 0 ms and ends at 100 ms. (B) Receptive fields of different neurons (σ=2). The receptive field range of a single neuron is $\pm 20^{\circ }$. (C) Receptive fields of different neurons (σ=3). The receptive field range of a single neuron is $\pm 30^{\circ }$. (D) Receptive fields of different neurons (σ=4). The receptive field range of a single neuron is $\pm 40^{\circ }$.

The neurons representing the angles in the M1 and V are described by Equation 1. $S_{j}\left(t\right)$ represents stimulus j at time *t*, and V_*j*_(t) represents the firing rate of the neuron. The intensity of a stimulus is related to the receptive field of the neuron. Equation 2 describes the receptive field of the $\theta_{j}$. The range of neuronal receptive fields is set by σ. The larger the σ, the larger the range of neuronal receptive fields and the lower the perception precision. The smaller the σ, the smaller the range of neuronal receptive fields and the higher the perception precision. When the stimulation angle was *J*, the stimulation intensity of the neuron representing *J* was the largest, and the stimulation intensities of the other neurons decreased successively. The firing rate of the neuron increased when the stimulus is present and decays when the stimulus ended. *C* is a parameter that controls the rates of increase and decrease. We set C=0.04 in the M1, V, S1, and EBA areas; C=0.01 in the TPJ area; and C=0.15 in the AI area. The firing rates of the neurons and the receptive fields of different neurons are shown in Figure 11.

(Equation 1) $$\Delta V_{j}\left(t\right)=-C\times \left(V_{j}\left(t\right)-S_{j}\left(t\right)\right)$$

(Equation 2) $$s=e^{\left(\theta -\theta_{J}\right)/2\sigma^{2}}$$

The neurons in other areas are described by Equation 3. $V_{i}$ is the firing rate of the postsynaptic neuron, $V_{j}$ is the firing rate of the presynaptic neuron, and $W_{ij}$ is the synaptic weight between the presynaptic neuron *j* and the postsynaptic neuron *i*. Specifically, the neurons in each brain area in the computational model are described as shown in Equation 4.

(Equation 3) $$\begin{array}{l} V_{i}\left(t+1\right)=V_{i}\left(t\right)+\Delta V_{i}\left(t\right)\\ \Delta V_{i}\left(t\right)=-C\times \left(V_{i}\left(t\right)-\tanh\left(W_{ij}\times V_{j}\left(t\right)\right)\right) \end{array}$$

(Equation 4) $$\begin{array}{l} V_{S1}\left(t+1\right)=V_{S1}\left(t\right)+\Delta V_{S1}\left(t\right)\\ \Delta V_{S1}\left(t\right)=-C\times \left(V_{S1}\left(t\right)-\tanh\left(W_{M1-S1}\times V_{M1}\left(t\right)\right)\right)\\ V_{EBA}\left(t+1\right)=V_{EBA}\left(t\right)+\Delta V_{EBA}\left(t\right)\\ \Delta V_{EBA}\left(t\right)=-C\times \left(V_{EBA}\left(t\right)-\tanh\left(W_{V-EBA}\times V_{V}\left(t\right)\right)\right)\\ V_{TPJ}\left(t+1\right)=V_{TPJ}\left(t\right)+\Delta V_{TPJ}\left(t\right)\\ \Delta V_{TPJ}\left(t\right)=-C\times \left(V_{TPJ}\left(t\right)-\tanh\left(W_{S1-TPJ}\times V_{S1}\left(t\right)+W_{EBA-TPJ}\times V_{EBA}\left(t\right)\right)\right)\\ V_{AI}\left(t+1\right)=V_{AI}\left(t\right)+\Delta V_{AI}\left(t\right)\\ \Delta V_{AI}\left(t\right)=-C\times \left(V_{AI}\left(t\right)-\tanh\left(W_{S1-AI}\times V_{S1}\left(t\right)+W_{TPJ-AI}\times V_{TPJ}\left(t\right)+W_{EBA-AI}\times V_{EBA}\left(t\right)\right)\right) \end{array}$$

### Synaptic plasticity algorithm

In this model, the synaptic plasticity is defined as shown in Equation 5. $W_{ij}$ represents the synaptic weight between the postsynaptic neuron *i* and the presynaptic neuron population *j*:

(Equation 5) $$W_{ij}\left(T+1\right)=W_{ij}\left(T\right)+\Delta W_{ij}\left(T+1\right)\times W_{inhibit}\left(T+1\right)$$

where

$$\Delta W_{ij}\left(T+1\right)=\int_{T}^{T+1}\Delta w_{ij}\left(t\right)dt$$

The $\Delta w_{ij}\left(t\right)$ is calculated using Equation 6:

(Equation 6) $$\Delta w_{ij}\left(t\right)=\alpha V_{i}\left(t\right)V_{j}\left(t\right)+\beta V_{i}^{\prime }\left(t\right)V_{j}\left(t\right)+\gamma V_{i}\left(t\right)V_{j}^{\prime }\left(t\right)$$

where

$$\alpha =\int_{-\infty }^{+\infty }f\left(u\right)du,\beta =\int_{-\infty }^{0}uf\left(u\right)du,\gamma =-\int_{0}^{+\infty }uf\left(u\right)du$$

f(u) is the spike-timing-dependent plasticity (STDP) function reported in.^37^^,^^38^^,^^39^ According to our previous research in,^40^ we set the parameters of α=−0.0035, β=0.35, γ=−0.55 in this model.

$W_{inhibit}$ is the lateral inhibitory synaptic weight used to control the location of synaptic weight update, which is described by Equation 7. fs is the firing state of the postsynaptic neuron, and fn is the number of firings of the postsynaptic neuron. When the firing rate of the postsynaptic neuron was greater than the threshold, the firing state of the neuron was 1; otherwise it was 0. In this model, we set the threshold as 0.7;

(Equation 7) $$W_{inhibit}\left(T+1\right)=\tanh\left(W_{inhibit}\left(T\right)-\frac{2\times \arccos\;fs}{\pi }\times e^{fn}-1\right)+1$$

where

$$fs=\left\{ \begin{array}{cc} 1 & V_{i}>threshold\\ 0 & V_{i}\leq threshold \end{array}\right.$$

### Synaptic weight setting

The synaptic weights between brain areas were set based on the functions of the brain areas in the computational model. The initial weights are all 1, indicating that they are all excitatory connections. Some of these synaptic weights were fixed, while others were acquired through learning. The fixed weights included $W_{M1-S1},W_{S1-TPJ},W_{TPJ-AI},W_{V-EBA},W_{EBA-TPJ}$, and the weights remained unchanged during the training process. The variable weights include $W_{S1-AI}$ and $W_{EBA-AI}$, which learn based on synaptic plasticity during training and exhibit excitatory or inhibitory connections. In our computational model, the S1 receives stimuli from the M1 for the perception of arm movement orientation, and the EBA receives information from V and obtains finger orientation information; the function of $W_{M1-S1}$ and $W_{V-EBA}$ is information transmission, so the $W_{M1-S1}$ and $W_{V-EBA}$ were fixed weights. The TPJ receives information from the S1 and EBA and performs primary multisensory information integration. Primary multisensory integration is the basis for multisensory integration, so $W_{S1-TPJ}$ and $W_{EBA-TPJ}$ are also set as fixed weights. The AI receives information from the S1, TPJ, and EBA; performs high-level multisensory information integration; and outputs the result. To receive the primary multisensory information from the TPJ, $W_{TPJ-AI}$ was set to a fixed weight. To realize flexible high-level multisensory integration, $W_{S1-AI}$ and $W_{EBA-AI}$ were set to variable weights, acquired through learning.

### Training process

In the computational model, all brain areas were composed of neurons representing different angles. During training and testing, the inputs and outputs of the model were angles.

In the robot experiment, (1) the robot randomly generates the angle $\theta_{j}$ and inputs it into M1, which is used to control the movement of the robot’s finger. Each neuron in M1 represents a different angle, and the receptive field of each neuron is shown in Equation 2. Each neuron in the M1 generates the corresponding stimulus according to Equation 2 and a firing rate according to Equation 1. (2) The robot collects image information through the cameras in its eyes, uses our previous methods of motion perception and pattern recognition^41^^,^^42^^,^^43^ to obtain the angle of its finger movement, and inputs it into V. As with the M1, each neuron in V represents a different angle, generating corresponding stimuli according to Equation 2 and a firing rate according to Equation 1. (3) Neurons in the S1, EBA, TPJ, and AI generate firing rates according to Equation 4. (4) Synaptic plasticity occurs between the S1 and AI and between the EBA and AI. The update of synaptic weights is realized according to the firing rate of neurons in the S1, EBA, and AI and Equation 5. The neurons in the S1 and EBA are presynaptic neurons *j*, and the neurons in AI are postsynaptic neurons *i*. (5) Due to the receptive field characteristics of neurons, when neurons in the brain receive a single-angle stimulus, multiple neurons will generate varying degrees of firing rate. Therefore, it will result in continuous updates of synaptic weights between multiple neurons, making the model unable to converge. We implemented a local update of synaptic weights by using Equation 7 to update the synaptic weights of neurons that fired earlier and for a longer period of time.

In the simulation environment, the training process of the model was consistent with the robotics experiments. More details regarding the training and testing processes can be found in the open source code.
